# Supplementary material for: COVID-19 Preventive Behaviours in Cameroon: A Six-Month Online National Survey
Source: Int J Environ Res Public Health. 2021 Mar 4;18(5):2554. doi: 10.3390/ijerph18052554 (PMC7967528; doi:10.3390/ijerph18052554)
Supplement: Supplementary file 1 [file ijerph-18-02554-s001.pdf]

Supplementary Material:

Table 1. Data disaggregated by region for the preventive measures included in adherence score.

	Physical distancing	Hand hygiene	Mask use*	Cover mouth when coughing	Avoid touching face	Score: 0	Score: ≥3
Centre (*n*=2193)	1420 (63.6%)	2087 (93.5%)	1890 (85.8%)	1700 (77.5%)	1473 (67.2%)	12 (0.55%)	1897 (86.5%)
Adamawa (*n*=319)	231 (71.5%)	309 (95.7%)	290 (90.3%)	250 (78.4%)	230 (72.1%)	0 (0%)	284 (89.0%)
East (*n*=181)	129 (70.9%)	178 (97.8%)	162 (90.5%)	135 (74.6%)	133 (73.5%)	1 (0.55%)	169 (93.4%)
Far North (*n*=425)	283 (64.9%)	410 (94.0%)	373 (87.1%)	332 (78.1%)	286 (67.3%)	1 (0.24%)	380 (89.4%)
Littoral (*n*=1764)	1180 (65.4%)	1694 (94.0%)	1534 (86.4%)	1414 (80.2%)	1149 (65.1%)	1 (0.06%)	1535 (87.0%)
North (*n*=237)	167 (68.7%)	231 (95.1%)	210 (86.8%)	180 (75.9%)	164 (69.2%)	0 (0%)	211 (89.0%)
North West (*n*=731)	545 (72.7%)	723 (96.4%)	709 (95.2%)	673 (92.1%)	524 (71.7%)	1 (0.14%)	668 (91.4%)
South (*n*=235)	167 (70.2%)	226 (95.0%)	218 (92.0%)	185 (78.7%)	150 (63.8%)	0 (0%)	213 (90.6%)
South West (*n*=723)	520 (71.0%)	675 (92.2%)	654 (91.1%)	665 (92.0%)	477 (66.0%)	3 (0.41%)	632 (87.4%)
West (*n*=573)	373 (62.4%)	560 (93.6%)	512 (87.4%)	432 (75.4%)	392 (68.4%)	1 (0.17%)	508 (88.7%)
χ^2 p-value	*< 0.001*	*0.0156*	*< 0.001*	*< 0.001*	*0.0237*	*0.157*	*0.007*
**92 Missing values*							
